# Differences in problem behaviour among ethnic minority and majority preschoolers in the Netherlands and the role of family functioning and parenting factors as mediators: the Generation R Study

**DOI:** 10.1186/1471-2458-12-1092

**Published:** 2012-12-19

**Authors:** Ilse JE Flink, Pauline W Jansen, Tinneke MJ Beirens, Henning Tiemeier, Marinus H van IJzendoorn, Vincent WV Jaddoe, Albert Hofman, Hein Raat

**Affiliations:** 1 The Generation R study group, Erasmus University Medical Centre, Rotterdam, The Netherlands; 2 Department of Public Health, Erasmus University Medical Centre, P.O. Box 2040, Rotterdam, CA 3000, The Netherlands; 3 Department of Child & Adolescent Psychiatry, Erasmus University Medical Centre, Rotterdam, The Netherlands; 4 Department of Paediatrics, Erasmus University Medical Centre, Rotterdam, The Netherlands; 5 School of Pedagogical and Educational Sciences, Leiden University, Leiden, The Netherlands; 6 Department of Epidemiology, Erasmus University Medical Centre, Rotterdam, The Netherlands

**Keywords:** Ethnicity, Migration, Paediatric, Psychosocial factors, Mental Health

## Abstract

**Background:**

Studies have shown that, compared to native counterparts, preschoolers from ethnic minorities are at an increased risk of problem behaviour. Socio-economic factors only partly explain this increased risk. This study aimed to further unravel the differences in problem behaviour among ethnic minority and native preschoolers by examining the mediating role of family functioning and parenting factors.

**Methods:**

We included 4,282 preschoolers participating in the Generation R Study, an ethnically-diverse cohort study with inclusion in early pregnancy. At child age 3 years, parents completed the Child Behavior Checklist (CBCL/1,5-5); information on demographics, socio-economic status and measures of family functioning (maternal psychopathology; general family functioning) and parenting (parenting stress; harsh parenting) were retrieved from questionnaires. CBCL Total Problems scores in each ethnic subgroup were compared with scores in the Dutch reference population. Mediation was evaluated using multivariate regression models.

**Results:**

After adjustment for confounders, preschoolers from ethnic minorities were more likely to present problem behaviour than the Dutch subgroup (e.g. CBCL Total Problems Turkish subgroup (OR 7.0 (95% CI 4.9; 10.1)). When considering generational status, children of first generation immigrants were worse off than the second generation (P<0.01). Adjustment for socio-economic factors mediated the association between the ethnic minority status and child problem behaviour (e.g. attenuation in OR by 54.4% (P<0.05) from OR 5.1 (95% CI 2.8; 9.4) to OR 2.9 (95% CI 1.5; 5.6) in Cape Verdean subgroup). However, associations remained significant in most ethnic subgroups. A final adjustment for family functioning and parenting factors further attenuated the association (e.g. attenuation in OR by 55.5% (P<0.05) from OR 2.2 (95% CI 1.3; 4.4) to OR 1.5 (95% CI 1.0; 2.4) in European other subgroup).

**Conclusions:**

This study showed that preschoolers from ethnic minorities and particularly children of first generation immigrants are at an increased risk of problem behaviour compared to children born to a Dutch mother. Although socio-economic factors were found to partly explain the association between the ethnic minority status and child problem behaviour, a similar part was explained by family functioning and parenting factors. Considering these findings, it is important for health care workers to also be attentive to symptoms of parental psychopathology (e.g. depression), poor family functioning, high levels of parenting stress or harsh parenting in first and second generation immigrants with young children.

## Background

Studies have shown that, compared to native counterparts, preschoolers from ethnic minorities are at an increased risk of problem behaviour [1-3]. Studies aiming to explain this vulnerability have mostly focused on socio-economic influences and showed that more problem behaviour in ethnic minorities relative to the majority group were partly explained by income inequalities, poverty, low parental education, young and single parenthood [1,4]. Though socio-economic factors thus explain an important part of the association between the ethnic minority status and child problem behaviour, a substantial part of the association still remains unexplained.

Preschoolers have the family as a predominant environment, and as such the family exerts an important influence on their well-being. Family functioning and parenting factors have been found to vary between ethnic minority and majority groups, with ethnic minorities showing a greater risk of, amongst others, poor family functioning [5], parenting stress [6] and harsh parenting [7,8]. These differences can partly be explained by socio-economic status [9]. Additionally, factors unrelated to socio-economic status like migration and acculturation stress may also contribute to these differences [10].

A few studies have focused on how family factors contribute to the presence of problem behaviour in ethnic minority children [11-13]. Weiss et al. [11] demonstrated that the family’s reliability on internal coping strategies was a risk factor for problem behaviour in Latino children residing in the US. Varela et al. [14] showed that the influence of parental control and acceptance on anxiety symptoms differed between Latin-American, European- American and Mexican-American children.

Although the above studies provide important insights into how family factors contribute to problem behaviour in ethnic minorities, these studies do not unravel whether family functioning and parenting factors explain the ethnic differences in child problem behaviour and, whether this potential mediation is independent of socio-economic factors.

The present study sought to address this gap. The objectives were to investigate (1) whether problem behaviour at 3 years differs between children born to a Dutch mother and ethnic minority children; and (2) whether maternal psychopathology, family functioning, parenting stress, and harsh parenting mediate the association between the ethnic minority status and child problem behaviour. As acculturation levels may vary between first and second generation immigrants [15], we additionally investigated whether problem behaviour and potential mediating roles of family functioning and parenting factors differed according to maternal generational status. Our hypotheses were that (1) ethnic minority children, particularly those with first generation immigrant mothers, would present more problem behaviour than children born to Dutch mothers; (2) family functioning and parenting factors would partly mediate this association; and (3) mediation by family functioning and parenting factors will be stronger in children of first generation immigrants than in children of second generation immigrants due to family effects of migration stress [15].

## Methods

### Participants

This study was embedded in the Generation R study, a prospective population-based cohort from foetal life onwards in Rotterdam, the Netherlands [16]. The Medical Ethics Committee of the Erasmus MC, Rotterdam, approved this study. Written informed consent was obtained from all participants. All information that enabled identification of participants was excluded before distribution to the researchers [17].

**Figure 1:**
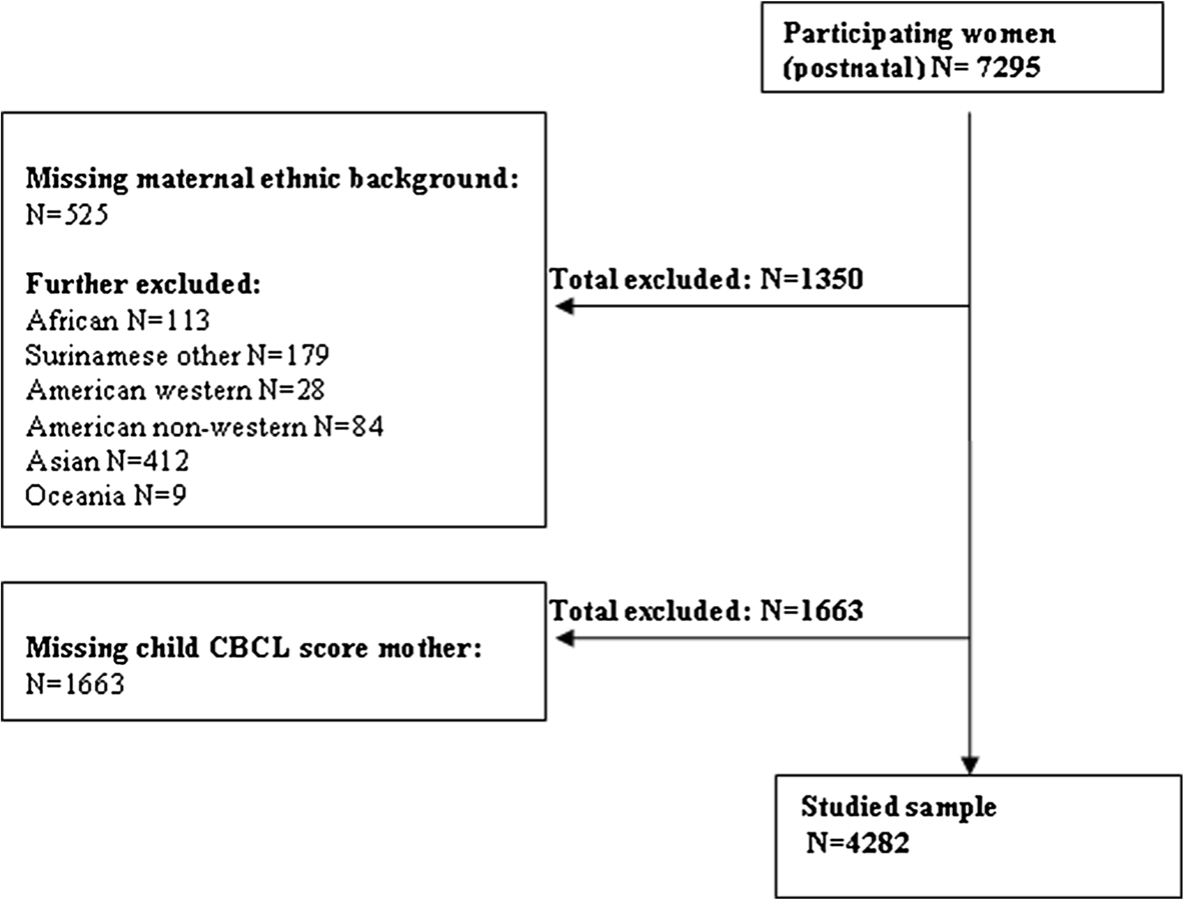
flowchart of the study population.

Full consent for the postnatal phase was obtained from 7295 participants. Mothers with missing data on their ethnic background (N=525) were excluded. Due to small numbers, classification difficulties or heterogeneity of groups, 825 mothers of different ethnic backgrounds were also excluded (i.e. Africans N=113, Surinamese other N=179, American Western=28, American non-Western=84, Asians N=412 and Oceania N=9). Children with no CBCL score (N=1663) were further excluded leaving 4282 children for analysis (see Figure 1).

### Measures

Data for this study were retrieved from medical records, and collected by prenatal and postnatal questionnaires. On request (i.e. in the case of illiteracy or very low education), trained research assistants with varied ethnic backgrounds helped with completing the questionnaires.

#### Ethnic background

We classified children according to maternal ethnic background. A choice was made for maternal ethnic background because mothers play an important role in young children’s lives and their ethnic background and experiences of acculturation are most likely to influence family functioning and parenting as well as child problem behaviour [18,19]. Maternal ethnic background was determined by the country of birth of the mother and the mother’s parents, a classification employed by Statistics Netherlands [20]. If the mother or one of her parents was born outside the Netherlands, this country of birth determined the ethnic background. If both parents were born outside the Netherlands, the country of birth of the mother’s mother determined the ethnic background. Women with a Surinamese background were further classified as Surinamese Hindu or Surinamese Creole. Subgroups of children in the study were: Dutch (N=3105), Other European (N=397), Antillean (N=78), Cape Verdean (N=94), Surinamese Hindu (N=85), Surinamese Creole (N=78), Moroccan (N=155) and Turkish (N=290). As a sensitivity analysis, we also considered paternal ethnic background for which a similar classification was employed. To account for differences in acculturation, we additionally established the generational status of non-Dutch participants. The first generation group included mothers who were born outside the Netherlands; the second generation group included mothers who were born in the Netherlands.

#### Problem behaviour

Mothers and fathers were both asked to fill out the Child Behavior Checklist (CBCL/1,5-5) when the child was 3 years. The CBCL/1,5-5, is a self-administered parent-report questionnaire that contains 99 problem items rated on a 3-point scale: 0 (not true), 1 (somewhat or sometimes true) and 2 (very true or often true). By summing the raw scores, seven syndrome scales (Emotionally Reactive, Anxious/Depressed, Somatic Complaints, Withdrawn, Sleep Problems, Attention Problems and Aggressive Behaviour) can be computed. The CBCL/1,5-5 also includes a Total Problems summary scale which was used for this study. A higher score on the Total Problems scale represents a higher severity. Good reliability and validity have been reported for the CBCL/1,5-5 [21]. The CBCL was available in Dutch, Turkish and English. The great majority (96.3%) filled in the Dutch version.

#### Potential confounders and mediators

Child birth weight, gestational age at birth (≤36 weeks or >36 weeks), sex and age were treated as confounders in this study [1,22].

Based on previous studies [1,23,24], we treated the following socio-economic factors as mediators: maternal age; marital status (married/cohabiting or no partner); parity, maternal education, classified as ‘low’ (primary school, lower vocational training, intermediate general school, 3 years general secondary school), ‘medium’ (>3 years general secondary school; intermediate vocational training; 1st year higher vocational training), and ‘high’ (higher vocational training, Bachelor’s degree, higher academic education and PhD); family income was defined by the total net month income of the household and classified as ‘<1200 €’ (below social security level), ‘1200–2000 €’ and ‘>2000 €’ (more than modal income).

Measures of family functioning that were included as potential mediators were maternal psychopathology, assessed prenatally and two months postpartum with the Brief Symptom Inventory [25] and overall family functioning, assessed prenatally with the twelve item General Functioning scale of the McMasters Family Assessment Device (FAD) [26].

Measures of parenting that were included as potential mediators were overall parenting stress measured at child age 18 months and assessed with the “Nijmeegse Ouderlijke Stress Index-Kort” (NOSIK) [27], the Dutch version of the Parenting Stress Index-Short Form and, harsh parenting measured at child age 3 years and assessed through separate maternal and paternal self-reports based on the Parent–child Conflict Tactics Scale [28]. In a previous study, a factor analysis was conducted to identify harsh parenting items [7].

Internal consistencies of family functioning and parenting scales were good (α >0.70) and only marginally satisfactory for maternal and paternal harsh parenting (α=0.63).

### Statistical analyses

To handle missing data in the covariates (i.e. confounders and potential mediators), multiple imputation was applied [29]. Five imputed datasets were generated using a fully conditional specified model, thus taking into account the uncertainty of the imputed values. In line with previous studies [30], imputations were based on the correlations between each variable for which missing values were observed (e.g. maternal education) and other relevant participant characteristics.

Frequency tables and cross tabulations were used to explore characteristics of the study population (Table 1). Because the CBCL Total Problems scores were skewed and could not be transformed to satisfy the assumption of normality, we firstly dichotomized the scores according to the 83rd percentile borderline cut-offs of a Dutch reference population [31]. Hereafter we used a multivariable logistic regression (model 1; basic model) to examine the association between maternal ethnic background and maternal-reported CBCL total problems, adjusted for confounders (Table 2).

**Table 1:** Characteristics of participants

	**N**	**Dutch (ref) N=3105**	**European other N=397**	**Antillean N=78**	**Cape Verdean N=94**	**Moroccan N=155**	**Surinamese Creole N=78**	**Surinamese Hindu N=85**	**Turkish N=290**	**p-value**
**Child characteristics**										
Sex (% boys)	4186	50.2	46.5	46.2	46.8	49.7	57.9	40.0	50.2	0.35
Age (months)	4282	36.5 (1.2)	36.6 (1.1)	37.1 (2.4)	36.9 (1.4)	37.2 (2.0)	36.8 (1.4)	36.9 (1.6)	37.2 (1.8)	<0.001
Birth weight (grams)	4184	3511.2 (551.4)	3468.8 (539.0)	3196.0 (520.9)	3247.1 (563.0)	3483.7 (518.1)	3254.7 (559.5)	3067.7 (476.9)	3391.8 (519.5)	<0.001
Gestational age at birth (%≤36 weeks)	4282	4.6	5.3	9.0	1.1	3.9	3.8	4.7	4.8	0.46
**Socio-economic characteristics**										
Age mother at intake (years)	4282	32.2 (4.0)	31.6 (4.3)	28.3 (5.4)	29.6 (5.3)	28.9 (5.1)	30.9 (5.9)	28.7 (5.4)	28.2 (5.3)	<0.001
Educational level	4212									
High (%)		66.9	66.6	25.6	12.5	16.0	23.7	20.2	15.0	<0.001
Medium (%)		31.7	28.4	65.4	64.8	57.6	65.8	69.0	49.1	
Low (%)		1.4	5.0	9.0	22.7	26.4	10.5	10.7	35.9	
Marital status (% single)	4187	5.0	5.6	40.3	40.0	5.3	44.9	22.4	5.3	<0.001
Family income	3584									
>2000 (%)		85.6	77.1	27.9	16.9	18.7	34.5	37.1	21.3	<0.001
1200-2000 (%)		11.4	16.8	31.1	33.8	38.3	31.0	30.0	40.0	
<1200 (%)		3.0	5.5	41.0	49.4	43.0	34.5	32.9	38.7	
Parity (% nulli)	4167	60.3	62.0	67.5	42.9	40.5	55.8	56.5	46.6	<0.001
**Family functioning and parenting characteristics**										
Prenatal maternal psychopathology ^1^	3435	0.1 (0.2)	0.2 (0.2)	0.2 (0.5)	0.3 (0.7)	0.3 (0.5)	0.2 (0.3)	0.3 (0.4)	0.4 (0.6)	<0.001
Postnatal maternal psychopathology ^1^	3732	0.1 (0.2)	0.1 (0.2)	0.2 (0.3)	0.2 (0.4)	0.2 (0.4)	0.2 (0.2)	0.2 (0.5)	0.3 (0.5)	<0.001
Prenatal family functioning ^1^	3838	1.3 (0.6)	1.4 (0.7)	1.7 (0.8)	1.9 (0.4)	1.8 (0.8)	1.6 (0.8)	1.9 (0.7)	1.7 (0.8)	<0.001
Parenting stress ^1^	3817	0.2 (0.3)	0.3 (0.4)	0.3 (0.3)	0.3 (0.5)	0.3 (0.4)	0.2(0.3)	0.3(0.4)	0.5(0.5)	<0.001
Harsh parenting (above cut-off)										
Maternal report (%)	3543	14.6	19.3	30.4	22.7	24.7	19.0	27.0	16.8	<0.001
Paternal report (%)	4251	13.2	22.1	23.7	23.4	18.7	21.1	27.7	18.5	<0.001

Values are percentages for categorical variables, means (sd) for continuous, normally distributed variables and medians (IQD) for non-normally distributed variables.

^1^ Median (IQD).

**Table 2:** Adjusted associations between maternal ethnic background and maternal-reported Total Problems

**Maternal ethnic background**	**Model 1**	**Model 2**	**% change**^**a**^	**Model 3**	**% change**^**b**^	**Model 4**	**% change**^**b**^	**Model 5**	**% change**^**b**^	**Model 6**	**% change**^**b**^	**Model 7**	**% change**^**b**^	**Model 8**	**% change**^**b**^
Dutch N=3105	1.0	1.0		1.0				1.0		1.0		1.0		1.0	
European other N=397	2.4	2.2		2.0		2.0		2.1		1.9		2.0		1.5	
	(1.6; 3.7)	(1.5: 3.5)	−14.4%^*^	(1.3; 3.0)	−22.2%^*^	(1.3; 3.1)	−22.2%^*^	(1.4; 3.3)	−10.1%^*^	(1.2; 3.0)	−26.4%^*^	(1.3; 3.1)	−19.5%^*^	(1.0; 2.4)	−55.5%^*^
Antillean N=78	4.2	2.6		2.5		2.6		2.6		2.5		2.3		2.1	
	(2.1; 8.4)	(1.2; 5.5)	−49.1%*	(1.2; 5.2)	−10.1%	(1.2; 5.6)	+0.2%	(1.2; 5.4)	−3.3%	(1.1; 5.4)	−9.1%	(1.1; 4.9)	−19.5%	(1.0; 4.7)	−29.4%
Cape Verdean N=94	5.1	2.9		2.4		2.7		2.5		3.0		2.6		2.3	
	(2.8; 9.4)	(1.5: 5.6)	−54.4%^*^	(1.2; 4.8)	−23.3%	(1.4; 5.3)	−5.8%	(1.3; 4.9)	−20.3%^*^	(1.5; 5.9)	+4.7%	(1.3; 5.1)	−15.1%	(1.1; 4.6)	−30.3%
Moroccan N=155	3.8	2.4		1.9		2.0		2.2		2.3		2.4		1.8	
	(2.2; 6.5)	(1.3; 4.4)	−49.2%^*^	(1.0; 3.4)	−39.8%^*^	(1.1; 3.8)	−22.0%^*^	(1.2; 3.9)	−18.3%^*^	(1.2; 4.2)	−9.9%	(1.3; 4.3)	−4.1%	(1.0; 3.4)	−43.8%^*^
Surinamese Creole N=78	1.9	1.2		1.2		1.3		1.1		1.3		1.1		1.1	
	(0.8; 4.9)	(0.5; 3.2)	−77.2%	(0.4; 3.2)	+8.8%	(0.5; 3.6)	+60.2%	(0.4; 3.0)	−38.9%	(0.5; 3.4)	+24.3	(0.4; 2.9)	−67.7%	(0.4; 2.9)	−73.9%
Surinamese Hindu N=85	6.8	4.7		4.2		3.9		3.9		4.6		4.0		3.3	
	(3.7; 12.3)	(2.5; 8.8)	−35.8%^*^	(2.2: 8.0)	−14.0%	(2.0; 5.6)	−19.7%^*^	(2.1; 7.5)	−20.5%^*^	(2.4; 8.9)	−2.5%	(2.1; 7.6)	−20.7%^*^	(1.7; 6.6)	−37.3%^*^
Turkish N=290	7.0	4.2		3.2		3.1		3.9		3.1		4.2		2.6	
	(4.9; 10.1)	(2.7; 6.6)	−46.4%^*^	(2.0; 5.0)	−32.4%^*^	(2.0; 4.9)	−31.5%^*^	(2.5; 6.1)	−10.1%^*^	(2.0; 4.9)	−35.1%^*^	(2.6; 6.5)	−2.7%	(1.6; 4.1)	−51.3%^*^

Table based on imputed dataset.

Values are OR (95% CI) derived from logistic regression models modelling maternal ethnic background as the determinant and maternal-reported Total Problems as the outcome variable.

Model 1: Basic model adjusted for child gestational age, birth weight, age, gender.

Model 2: Model 1+ maternal age, maternal marital status, maternal educational level, parity and family income.

Model 3: Model 2 + prenatal maternal psychopathology.

Model 4: Model 2 + postnatal maternal psychopathology.

Model 5: Model 2+ prenatal family functioning.

Model 6: Model 2+ parenting stress.

Model 7: Model 2 + paternal harsh parenting.

Model 8: Fully adjusted model.

^a^ Change in odds ratio relative to model 1 for non-Dutch ethnic groups versus Dutch reference group (100 * [OR_model 1+mediator_– OR_model 1_] / [OR_model 1_ -1])).

^b^ Change in odds ratio relative to model 2 for non-Dutch ethnic groups versus Dutch reference group after individual adjustment (models 3–7) or full adjustment

(model 8) for family functioning and parenting factors (100 * [OR_model 2+mediator_– OR_model 2_] / [OR_model 2_ -1])).

^*^ p <0.05 indicates a significant change in odds ratio after adding variable(s) to model 1 or model 2 calculated with a bootstrap analysis.

Some of the family functioning and parenting factors were also skewed and were transformed (using the square root and the natural log) to approach normality. Harsh parenting could not be normalized and was therefore dichotomized. The 20*%* highest scoring mothers and fathers were considered as parents who use harsh parenting.

**Figure 2:**
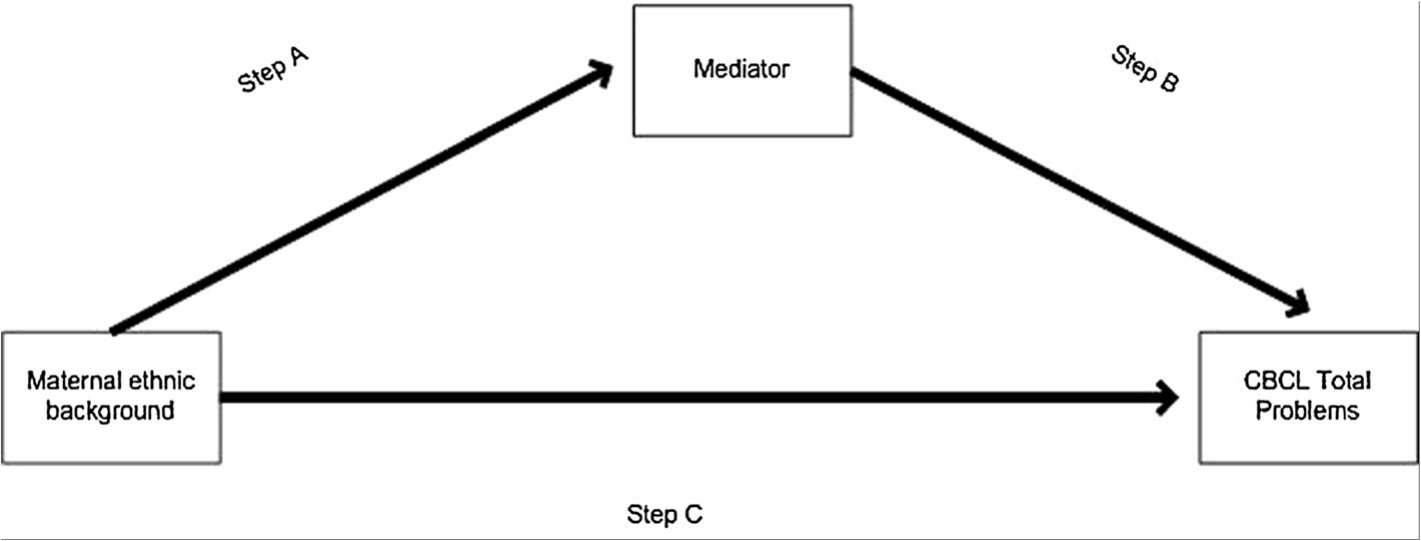
Causal step approach for the selection of mediators.

We assessed mediation of the family functioning and parenting factors by following the causal step approach proposed by Baron and Kenny (Figure 2) [32].

We conducted a series of regression models to test (1) the association between maternal ethnic background and potential mediators (data not shown; Step A) and (2) the association between the potential mediators and CBCL Total problems adjusted for maternal ethnic background (Additional file 1: Table S2; Step B). Factors that were significantly associated with maternal ethnic background and CBCL Total Problems were considered ‘true’ mediators and were selected for a third and final step (Step C). In this step, we separately added the mediators to model 1 to evaluate the attenuation (or increase) of the original association of maternal ethnic background with CBCL Total Problems (Table 2). Model 2 included the confounders and socio-economic factors. Hereafter, the family functioning and parenting factors were individually added to model 2 (models 3–7). Finally, the 8^th^ model was the ‘full’ model including confounders, socio-economic and family functioning and parenting factors. The mediating roles of the socio-economic and family functioning and parenting factors were assessed by calculating the percentage change in Odds Ratio (OR) relative to model 1 (socio economic factors) or, model 2, (family functioning and parenting factors) (e.g. (100 * [OR_model 1+mediator_– OR_model 1_ / [OR_model 1_ -1])). Additionally a bootstrap analysis was conducted to test the whether the strength of the association changed after addition of the mediators [33].

To assess whether results changed if we included paternal-reported Total Problems as the outcome or paternal ethnic background as the determinant, we separately repeated the analyses with this outcome and determinant (data not shown). We additionally repeated the analyses with maternal generational status (first or second generation) as the main determinant and maternal-reported Total Problems as the outcome (Table 3).

**Table 3:** Adjusted associations between maternal generational status and maternal-reported Total Problems

	**Model 1**	**Model 2**	**% change**^**b**^	**Model 3**	**% change**^**c**^
Dutch N=3105	1.0	1.0		1.0	
First generation immigrants N=835	5.0 (3.8; 6.8) ^a^	3.3 (2.3; 4.6)	−44.6%^*^	2.3 (1.6; 3.2)	−44.5%^* d^
Second generation immigrants N=317	2.5 (1.6; 4.0)	1.8 (1.1; 3.0)	−47.0%^*^	1.3 (0.8; 2.1)	−66.0%^*^

Table based on imputed dataset.

Values are OR (95% CI) derived from logistic regression models modelling maternal generational status as the determinants and maternal-reported Total Problems as the outcome variable.

Model 1: Basic model adjusted for child gestational age, birth weight, age and gender.

Model 2: Model 1+maternal age, maternal marital status, maternal educational level, parity and family inome.

Model 3: Model 2+ prenatal maternal psychopathology + postnatal maternal psychopathology + prenatal family functioning + parenting stress + paternal harsh parenting.

^a^ p<0.01 for first generation vs. second generation (reference).

^b^ Change in odds ratio relative to model 1 for non-Dutch ethnic groups versus Dutch reference group (100 * [OR_model 1+mediator_– OR_model 1_] / [OR_model 1_ -1])).

^c^ Change in odds ratio relative to model 2 for non-Dutch ethnic groups versus Dutch reference group after full adjustments (model 3) for family functioning and parenting.

factors (100 * [OR_model 2+mediator_– OR_model 2_] / [OR_model 2_ -1])).

^d^ P=0.26 for difference in odds ratio attenuation between first and second generation immigrants calculated with a bootstrap analysis.

^*^ p <0.05 indicates a significant change in odds ratio after adding variable(s) to model 1 or model 2 calculated with a bootstrap analysis.

#### Non-response analysis

A comparison of ethnic minority children included in this study (N=2158) with children who were excluded due to missing values for maternal ethnicity (N=525) did not indicate any significant differences in terms of maternal educational level, marital status and child problem behaviour. We also compared the ethnic minority children included in this study to children who were excluded due to ethnic classification difficulties and small sample sizes (N=825). We found that the excluded group was higher educated (X^2^=53.1; P<0.001) than the ethnic minorities that were included. The groups did not differ on marital status and child problem behaviour.

## Results

### Characteristics

Characteristics of the participants are presented in Table 1. Ethnic differences were present in almost all variables except for gestational age and gender. Ethnic differences were also found for family functioning and parenting factors e.g. paternal harsh parenting (X^2^=46.7; P<0.001).

### Maternal ethnic background and child problem behaviour

Compared to children born to a Dutch mother, children from six out of seven ethnic minorities had an increased risk of problem behaviour after adjustment for child gender, age, birth weight and gestational age (Table 2; model 1). The risk was the most increased in the Turkish subgroup (OR 7.0 (95% CI 4.9-10.1)).

### Mediation

All six family functioning and parenting factors that were considered potential mediators met Baron and Kenny’s [32] criteria for mediation. However, maternal harsh parenting was excluded as a mediator because the correlation with paternal harsh parenting was strong (r=0.40) and paternal harsh parenting was more strongly associated with ethnic background and CBCL Total Problems (Table 2). Hence, the five factors that we studied as mediators were prenatal and postnatal maternal psychopathology, prenatal family functioning, parenting stress at child age 1,5 years and paternal harsh parenting at child age 3 years.

Table 1 shows the adjusted associations between maternal ethnic background and CBCL Total Problems. Compared to the model adjusted for confounders, adjustment for socio-economic factors attenuated the association between ethnic background and CBCL Total Problems by up to 54.4% (Cape Verdean subgroup; P<0.05). Mediation by socio-economic factors was strong but partial as the associations between ethnic background and CBCL total problems were still significant in six out of seven ethnic subgroups. Compared to the model adjusted for confounders and socio-economic factors, individual adjustments for the family functioning and parenting factors resulted in up to 39.8 % attenuation in the OR. The mediating roles of the individual family functioning and parenting factors differed per ethnic minority group. For instance, adjustment for prenatal maternal psychopathology resulted in 39.8% (P<0.05) attenuation in the OR in the Moroccan subgroup while paternal harsh parenting was the strongest mediator in the Surinamese Hindu subgroup, accounting for 19.6% (P<0.05) attenuation in the OR. Adjustments for all family and parenting factors combined resulted in up to 55.5% (European subgroup; P<0.05) attenuations in the ORs.

We repeated the analyses with paternal reports of CBCL Total Problems (n=3568; data not shown). Results were very similar to maternal reports. We also repeated the analyses with paternal ethnic background (n=3254; data not shown), which also yielded similar results.

We assessed whether child problem behaviour differed between children of first and second generation immigrants compared to children classified as Dutch and whether family functioning and parenting factors mediated this association (Table 3). After adjustment for confounders, ORs for maternal-reported CBCL Total Problems compared to the Dutch subgroup were higher in children of first generation immigrants than in children of second generation immigrants and this difference was significant (P<0.01). Socio-economic factors mediated the association to the same degree in the first and the second generation (i.e. attenuation in OR by 44.6% (P<0.05) in the first generation and, attenuation in OR by 47.0% (P<0.05) in the second generation). After adjustment for confounders and socio-economic factors, family functioning and parenting factors additionally mediated the association in both generational groups (P<0.05). Although mediation appeared to be stronger in the second than in the first generation group this difference was not significant (P=0.26).

## Discussion

This large multi-ethnic population study showed that parents from non-Dutch ethnic minorities report more problem behaviour in their 3-year-old children than parents from the Dutch majority group. Although socio-economic factors explained a substantial part of this relationship, a similar part was explained by maternal psychopathology, family functioning, overall parenting stress and paternal harsh parenting.

Before discussing the findings of this study further, some methodological considerations need to be taken into account. A strength of this study is the large number of participants from different ethnic groups and the population-based design. A limitation is that we had to rely on parent-reports of problem behaviour as the children were too young for self-reports or assessments by teachers or other informants, and because it was not feasible to obtain clinical diagnoses in such a large sample of children. However, we did have maternal and paternal reports which yielded very similar findings. In this study, some children were excluded due to missing data on ethnic background, ethnic classification difficulties or small sample sizes of some ethnic groups. We demonstrated that the excluded children had slightly higher educated mothers than the ethnic minority children included in the study. However, as no differences were observed for other socio-economic characteristics and child problem behaviour, we do not think that non-response or the exclusion of small ethnic minority groups substantially influenced our findings. An additional limitation was that the direction of causation could not be determined for the postnatal mediators (overall parenting stress and harsh parenting). To partly address this issue we repeated the mediation analysis for overall parenting stress in a subsample of children who did not present behavioural problems at 18 months (n=3505; data not shown). Although the sample of children that presented problem behaviour at 36 months was substantially smaller, the findings were fairly similar to our initial findings. This substantiates the hypothesized causality of our model, that parental stress influences child problem behaviour rather than only being a consequence of it. However, as harsh parenting was measured at the same age as the outcome of our study, we were not able to check the assumed causal relation for this mediating variable. Children in this study were classified according to maternal ethnic background and some children may therefore have been misclassified. However, classifying children according to paternal ethnic background yielded very similar findings. Lastly, most of the family functioning mediators included in this study were measured during pregnancy to limit the possibility of reverse causality; that is child behaviour influencing family functioning rather than reverse. However, as a result these mediating factors were quite distal and were therefore limited in their mediating effect. Hence, future studies may want to also consider including family functioning factors measured closer to the outcome.

In the present study we found that children from non-Dutch ethnic minorities presented more problem behaviour than children born to a Dutch mother. When considering generational status, we found that the risk was particularly increased in children of first generation immigrants, though the second generation also presented more problem behaviour. A potential explanation for this finding is that immigration risk factors such poor proficiency of the native language and cultural barriers, more common in first than in second generation immigrants, can lead to social isolation and associated stress in mothers, which may affect children’s behaviour [1,34].

We additionally found that, besides socio-economic risk factors, differences in problem behaviour among ethnic minority preschoolers and preschoolers born to a Dutch mother could be explained by family risk factors like family functioning and parenting stress. There may be several explanations for this finding. Firstly, migration to a new country and culture often challenges familial roles and responsibilities and may also cause changes in family organisation and functioning [35-37]. Leidy et al. [12] for instance note that one of the challenges to positive parenting is a lack of extended family members who previously helped with raising children. Changes in family organisation and functioning may in turn lead to stress which, during pregnancy, can expose the foetus to elevated levels of stress hormones and possibly influence the development of stress systems [38]. After birth, maternal and family stress can influence parent–child interactions which has been associated with behavioural problems [39]. This is supported by our finding that both prenatal and postnatal maternal psychopathology mediated the association between the ethnic minority status and child problem behaviour.

It is also possible that family functioning and parenting factors are influenced by cultural norms and values related to ethnic background. Harsh parenting was for instance the strongest mediator in the Surinamese Hindu, Antillean and Cape Verdean subgroups. In these subgroups ‘machismo’, a cultural value characteristic that is particularly prominent in Latino and Caribbean populations and has been linked to harsh parenting, may partly explain this finding [10]. Additionally ‘familism’, a cultural value characteristic that is defined as “the subordination of individual interests to those of the family” [40], has also been linked to ethnic minorities [41]. Studies have shown that expectations of family harmony or ‘familism’ may create increased distress when conflicts within the family arise [5]. Cultural factors may also affect perceptions of a ‘normally’ functioning family, ‘harsh’ parenting and child behaviour. For instance, studies have shown that physical punishment is more accepted in some cultures than in others possibly leading to differences in the threshold to report harsh parenting [42].

In our study, we found that socio-economic factors mediated the association to the same degree in first and second generation immigrants. This indicates that socio-economic disadvantage affects the mental health of immigrant children in the Netherlands despite maternal generational status. Family functioning and parenting factors also explained the association between the immigrant status and problem behaviour in first and second generation immigrants. In contrast with our initial hypothesis, mediation appeared to be stronger in the second generation however, this difference was not significant. As acculturation levels may vary according to generational status and it is possible that this affects family functioning and parenting factors differently [43], we recommend further study into this issue.

## Conclusions

This study showed that preschoolers from ethnic minorities and particularly children of first generation immigrants are at an increased risk of problem behaviour compared to children born to a Dutch mother. Although socio-economic factors were found to partly explain the association between the ethnic minority status and child problem behaviour, a similar part was explained by family functioning and parenting factors. Considering these findings, it is important for health care workers to be attentive to symptoms of parental psychopathology (e.g. depression), poor family functioning, high levels of parenting stress and harsh parenting in first and second generation immigrants with young children. With proper screening, young immigrant parents may be able to receive intervention services that will not only serve to improve their own mental well-being, but also to help prevent the development of problem behaviour in their offspring. Ideally, such screening is done early in children’s lives, perhaps even before birth. Primary care doctors and nurses like general practitioners and professionals at well baby clinics, but also midwives and obstetricians might play a key role in the detection and referral of immigrant parents or parents-to-be who experience mental health problems.

## Competing interests

The authors have no competing interests to declare.

## Authors’ contribution

IF: Analysis, interpretation, drafting, reporting. PJ: Analysis, interpretation, revision. TB: Analysis, interpretation, revision. HT: Conception, design, interpretation, revision. RI: Conception, design, interpretation, revision. VJ: Conception, design, revision. AH: Conception, design, revision. HR: Conception, design, analysis, interpretation, revision. All authors contributed to the study design, analysis, interpretation of data or drafting or revision of the manuscript. All authors read and approved the final manuscript.

## Pre-publication history

The pre-publication history for this paper can be accessed here:

http://www.biomedcentral.com/1471-2458/12/1092/prepub

## Supplementary Material

Additional file 1 **Table S2.**Associations between family functioning and parenting factors and maternal-reported Total Problems (N=4282). Click here for file
