# Supplementary material for: Differences in problem behaviour among ethnic minority and majority preschoolers in the Netherlands and the role of family functioning and parenting factors as mediators: the Generation R Study
Source: BMC Public Health. 2012 Dec 19;12:1092. doi: 10.1186/1471-2458-12-1092 (PMC3577476; doi:10.1186/1471-2458-12-1092)
Supplement: Additional file 1 — Table S2.Associations between family functioning and parenting factors and maternal-reported Total Problems (N=4282). [file 1471-2458-12-1092-S1.doc]

Table S2 Associations between family functioning and parenting factors and maternal-reported Total Problems (N=4282)

| *Family functioning and parenting factors* | *OR (95% CI)* |
| --- | --- |
| Prenatal maternal psychopathology | 7.3 (4.4; 11.9) |
| Postnatal maternal psychopathology | 6.3 (4.0; 10.0) |
| Prenatal family functioning | 2.4 (1.8; 3.2) |
| Overall parenting stress | 17.3 (9.7; 31.1) |
| Maternal harsh parenting | 2.4 (1.8; 3.4) |
| Paternal harsh parenting | 2.9 (2.1; 3.8) |

Table based on imputed dataset.

Values are ORs (95% CI) derived from logistic regression models modelling family functioning and parenting factors as the determinant and maternal-reported Total Problems as the outcome variable, adjusted for maternal ethnic background.
